# 

**Published:** 1898-07

**Authors:** 


					﻿Vol. XVIII.
JULY, 1898.
NO. 7?
THE ' $-R
pOMEOPATHlC PHYSICIAN,
A Monthly Journal of Homoeopathic Materia Medica
and Clinical Medicine.
“ fie it a freeman wham truth makes free, and all are slaves beside.”
u Seek the Truth: come whence it may, cost what it will.”
BDITEDAND PUBLISH BD BY
WALTER MI. JAMES, ML D.
PHILADELPHIA :
ISTo. 1231 LOCTTST STREET.
Yearly Subscription, $2.50, invariably In advance. Single numb er s9 25 cis.
Entered at tbe Philadelphia Poat*Offlee a« Second-Cl aai Mail Matter.
				

